# Weight prediction in complex networks based on neighbor set

**DOI:** 10.1038/srep38080

**Published:** 2016-12-01

**Authors:** Boyao Zhu, Yongxiang Xia, Xue-Jun Zhang

**Affiliations:** 1 College of Information Science and Electronic Engineering, Zhejiang University, Hangzhou 310027, China; 2 School of Electronic and Information Engineering, Beihang University, Beijing 100191, China

## Abstract

Link weights are essential to network functionality, so weight prediction is important for understanding weighted networks given incomplete real-world data. In this work, we develop a novel method for weight prediction based on the local network structure, namely, the set of neighbors of each node. The performance of this method is validated in two cases. In the first case, some links are missing altogether along with their weights, while in the second case all links are known and weight information is missing for some links. Empirical experiments on real-world networks indicate that our method can provide accurate predictions of link weights in both cases.

Many real-world systems, such as social, biological and communication systems, can be described as networks, where nodes denote individuals and links represent interactions between them. Over the last few decades, the field of network science has been developed as a critical framework for understanding the organization of real networked systems^1^,^2^. Faithful representation of many real-world networks requires not only links to indicate the existence of interactions but also associated weights to express interaction strengths. Examples are ubiquitous. For example, in an airline network, the weight of a link may represent the number of flights^3^, the number of available seats^4^, or the number of passengers^5^ traveling between two airports. In food webs, link weights may represent energy or carbon flows between taxa^6^. In scientific collaboration networks, the weight of a link may quantify the number of papers co-authored by two researchers^7^.

Unfortunately, data collected from real-world networks are usually incomplete. This gives rise to two related data reconstruction problems. First, some links may be missing from the data, in which case we need to predict these missing links from the available data. This *link prediction* problem has received much attention in the past decade^8^,^9^,^10^,^11^,^12^, and many link prediction algorithms have been proposed for both unweighted^9^,^13^,^14^,^15^,^16^,^17^,^18^,^19^,^20^,^21^,^22^,^23^,^24^,^25^,^26^ and weighted^27^,^28^,^29^,^30^,^31^ networks. Second, the weights of some links may be unavailable. Of course, if the existence of a link is unknown, then its weight is obviously unavailable as well. However, even if the existence of a link is known, its weight may still be missing due to incomplete data, and in this case we need to estimate the missing weights. Unfortunately, only a few studies have focused on this second problem of *weight prediction*. Recently, Aicher *et al*. developed a weighted stochastic block model, that can be applied to infer both the existence and weights of links^29^. Zhao *et al*. proposed another method based on reliable routes to extend unweighted similarity indices to weighted ones^30^, which can be used to predict the weights of links by assuming that similarity scores are linearly correlated with link weights.

Although link prediction and weight prediction problems can be described by the same model, we believe it is better to separate these two tasks. First, they address different types of missing information, and therefore they CAN be separated. Second, according to the “no free lunch theorem”^32^, “*if an algorithm performs well on a certain class of problems then it necessarily pays for that with degraded performance on the set of all remaining problems*”. Based on this theory, separately designed link prediction and weight prediction algorithms should achieve better prediction performance than a single model for predicting both the existence and weights of links. Thus, we propose the following general process to predict the missing information. If links are missing from the data, we first perform link prediction and then predict the weights of the recovered links. If all of the links are available and the weights of only some links are missing, then we skip straight to weight prediction. The link prediction step of this process has been studied extensively, but the weight prediction step remains largely un-investigated. Therefore, in this paper we will focus on the essential problem of weight prediction.

In this study, we try to predict weights of links with the help of local structural information. We develop a novel method for predicting weights by examining the network structure surrounding a node, namely, its set of neighbors. The algorithm can be used in two cases, depending on whether there are missing links to be recovered or not. According to our assessments, the proposed method performs well in both cases. We hope this work may lead to a deeper insight into the design of weight prediction algorithms in weighted networks.

## Results

### Problem description

We are given an undirected weighted network *G*(*V*, *E*, *W*), where *V*, *E* and *W* denote the sets of nodes, links and link weights, respectively. Let *Adj* be the adjacency matrix of the network: if two nodes, say *i* and *j*, are connected, then we have *a*_*ij*_ = 1, where *a*_*ij*_ is the (*i*, *j*) entry of *Adj*; otherwise, *a*_*ij*_ = 0. Because the networks we considered here are undirected, the entries *w*_*ij*_ and *w*_*ji*_ of *W* are the same.

Usually, the incomplete information of networks, namely the missing links and their weights, is not available simultaneously. In this case, the prediction consists of two steps: link prediction and weight prediction. In the first step, our goal is to estimate the likelihoods of the existence of links in a given network. To do this, we assign a score, *s*_*xy*_, to each candidate node pair (*x*, *y*) ∈ *U* − *E*, where *U* stands for the universe of possible links to quantify the likelihood that the node pair (*x*, *y*) is connected, with a higher score indicating a more likely connection. Then, all candidate pairs are sorted by their scores in decreasing order, so that the most likely existed links are those with the highest ranks. More details about link prediction will be given in the Methods section. In the second step, we predict the weights for the highest-ranked links.

In the case where all the links are available and only the weights of some links are missing, we need to perform only the second step, *i*.*e*., weight prediction.

### A weight prediction method based on neighbor set

Our method relies on the assumption that the formation of link weights is regulated by local clusterings in which homogenous links tend to have similar weights. The local structure we considered is the neighbor set of a node, defined as the set of nodes linked to it, which captures a great deal of information about the node. For instance, in online social networks, the neighbors of a node represent the friends of a person. The co-existence of two people in the same neighbor set enhances the probability of their relationship changing from non-friends to friends.

**Figure 1:**
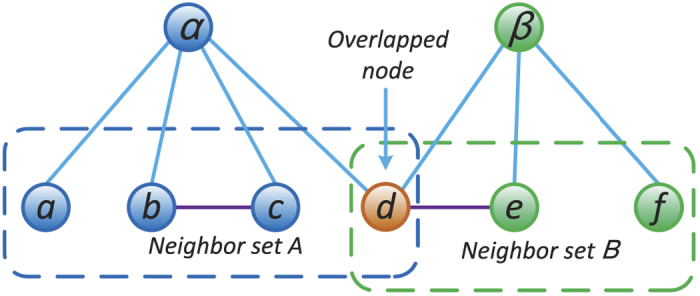
An illustration of neighbor set. In this example, neighbor set *A* is defined as the set of neighbors of node *α*, which are *a*, *b*, *c* and *d*. Neighbor set *B* consists of three nodes, namely, *d*, *e* and *f*. Note that node *α* and node *β* have one common neighbor, which belongs to both neighbor sets *A* and *B*. Within neighbor set *A*, because there is only one link, the existence probability for the remaining possible links is 
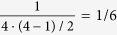
, while the remaining links in neighbor set *B* exist with probability 
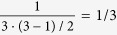
. Connections across neighbor sets *A* and *B* exist with probability 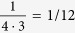.

Our method of weight prediction can be well explained by considering a simple example, illustrated in Fig. 1. If a link is generated between nodes *a* and *b*, then one wish to have a guess of the weight, *w*_*ab*_, of link (*a*, *b*). Since nodes *a* and *b* appear together in neighbor set *A*, according to our fundamental hypothesis, the weight of (*a*, *b*) is related to the weights of other similar links in *A*. Because only one other link (*b*, *c*) exists, here we focus on examining its relationship to the candidate link. From our perspective, the weight of (*b*, *c*) is correlated with the weights of links to common node *α*. For example, if Fig. 1 is an illustration of social networks and weights of the network indicate time commitments, then the amount of time *b* spends with *c* depends on the time *α* spends with *b* and *c*^33^. If the events “*α* is with *b*” and “*α* is with *c*” are independent of each other, then the event “*b* is with *c*” would have probability equal to the product of their probabilities. Based on this, one can simply estimate *w*_*ab*_ as 
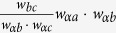
. If other similar links exist, an averaging strategy will be applied to combine the estimates. Similarly, one can use the weight of link (*d*, *e*) to infer the weight of link (*a*, *f*) across neighbor sets *A* and *B* by estimating the value of *w*_*af*_ to be 
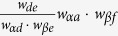
.

In practice, a node often belongs to more than one neighbor set. For example, in Fig. 1, node *d* belongs to both neighbor sets *A* and *B*. Indeed, any two candidate nodes may coexist in multiple neighbor sets. In this case, a natural approach is to calculate the individual contributions of different neighbor sets or pairs of neighbor sets to the candidate node pair and then combine them to obtain a more accurate weight. Viewed in terms of link formation, our hypothesis is that loosely connected clusterings are less likely than densely connected clusterings to form a link^34^ and thus contribute less to the link weight of the link once it is formed. Under this assumption, a more refined weight can be estimated based on the connection probabilities in or across different neighbor sets.

A detailed explanation is as follows. Suppose our goal is to estimate the weight of the link (*x*, *y*). Let Γ(*x*) be the set of neighbors of node *x* and 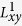 be the event that nodes *x* and *y* are connected. If nodes *x* and *y* both belong to the neighbor set of node *α*, *i*.*e*., *x*, *y* ∈ Γ(*α*), the weight of (*x*, *y*) can be written as


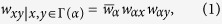


where


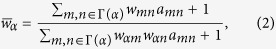


which is the average clustering weight over links similar to (*x*, *y*). Note that we apply *add*-*one smoothing* to preclude the possibility of an undefined fraction. Based on our hypothesis, in order to quantify the contribution of the neighbor set Γ(*α*) to the formation of *w*_*xy*_, we need to calculate the probability that the pair (*x*, *y*) is connected, given that both are in the neighborhood Γ(*α*). This probability can be estimated through


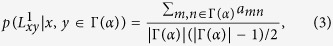


where |·| denotes the number of elements in the set. In fact, Eq. (3) is the clustering coefficient of node *α*, given by the link density within the neighbor set Γ(*α*).

On the other hand, if nodes *x* and *y* belong to different neighbor sets, say *x* ∈ Γ(*α*), *y* ∈ Γ(*β*), the weight of (*x*, *y*) can be described as


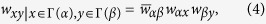


where


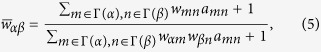


which is the average weight across clusterings. When nodes *x* and *y* appear in separate neighborhoods, we can use the connection probability across the two neighbor sets to measure their contribution to the formation of *w*_*xy*_. Then the probability that nodes *x* and *y* are connected can be written as


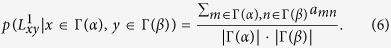


Clearly, this equation measures the connection density across neighbor sets Γ(*α*) and Γ(*β*).

Finally, by considering the contributions of different neighbor sets to the formation probability of the link (*x*, *y*), we can estimate *w*_*xy*_ by


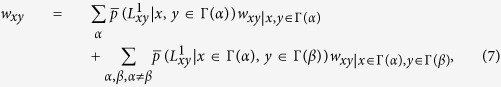


where 
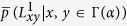
 and 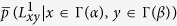 are normalized probabilities, defined as


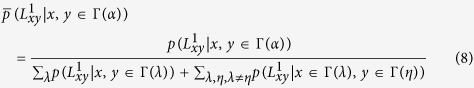


and


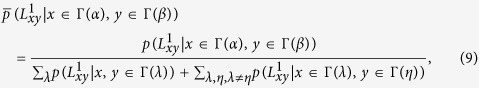


respectively.

### Experimental results

First, we consider the case where information is missing regarding both existence and weights of links. To validate the prediction accuracy, the observed links are randomly divided into two parts: training set *E*^*T*^ and validation set *E*^*V*^, where *E*^*T*^ is regarded as the given information, and *E*^*V*^ is only used for testing. Clearly, we have 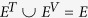 and 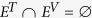. In this experiment, the training set contains 90% of the links, and the validation set contains the remaining 10%. With the help of link predictors, the candidate node pairs are sorted based on their scores. Then the top-*L* links are selected as the predicted link set *E*^*L*^. In this paper we set *L* as the size of validation set for the reason of weight prediction. After link prediction, the weight prediction algorithm is conducted. The corresponding predicted weight set and actual weight set are denoted as 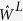 and *W*^*L*^, respectively. The actual weights for non-observed links are set to zero. In most cases, this default value is reasonable. For example, in transportation networks, if there is no connection between two nodes, then the traffic flow directly between these two nodes is zero. For some special cases, this is not the appropriate default, such as for those networks whose weights denote distances between nodes. However, this default is appropriate for all networks we presented here. Then the accuracy of weight predictor can be estimated by calculating the Pearson correlation coefficient and root mean squared error (RMSE) between the vectors 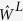 and *W*^*L*^.

Table 1 compares the accuracy of the linear-correlation method^30^ (refer to the Methods section for details) with that of our method, as measured by the Pearson correlation coefficient under different link prediction approaches. Each Pearson correlation coefficient is calculated between the vectors of predicted and actual weights for the top-*L* ranked links. A larger correlation coefficient indicates more accurate linear correlation between predicted and actual weights. As shown in the table, almost all of the correlation coefficients achieved by our method are larger than those from the linear-correlation method under every link prediction algorithm, indicating that the good performance is due to our method itself, regardless the detailed link prediction algorithms. The linear-correlation method assumes that weights of links measure similarities or affinities between nodes, so the correlation between similarities and weights is weak for those networks whose weights don’t exhibit similarities between nodes, such as the Everglades network. On such networks the linear-correlation method performs poorly. In contrast, our method can be applied to a wider range of networks, in which weights do not necessarily characterize similarities between nodes.

We also calculate the RMSE between the vectors of predicted and actual weights for the top-*L* ranked links. Detailed results are summarized in Table 2. As shown in the table, in most networks the weights predicted by our method have remarkably smaller errors than those predicted by the linear-correlation method under a variety of link prediction algorithms. In Everglades and USAir1, our method performs similarly to the linear-correlation method as measured by RMSE. However, combining with the metric of Pearson correlation coefficient, we can find that our method performs significantly better than the linear-correlation method on those networks. Furthermore, the linear-correlation method employs the information from the validation set to estimate the scaling coefficient in Eq. (19). As a result, predictive information from the validation set leaks into the optimization step and will lead to optimistically biased performance estimates^35^. This does not happen in our method because only the information from the training set is used.

Next, we consider the case where only the weight information is missing. In this case, we can directly set *E*^*L*^ as *E*^*V*^. For our method, the link prediction is not needed, and we can directly perform weight prediction. However, since the linear-correlation method uses link prediction to calculate the similarity scores *S*^*V*^, it actually still needs to perform both link prediction and weight prediction.

The Pearson correlation coefficient and RMSE between the vectors of predicted weights and actual weights are presented in Tables 3 and 4, respectively. Compared with the linear-correlation method, our method generally gives better estimates of weights in most networks. On the other hand, the advantages of our method are not so apparent when using RMSE to measure accuracy.

Altogether, empirical experiments indicate that the weights of links can be recovered more correctly by our method, in contrast to the linear-correlation method.

**Figure 2:**
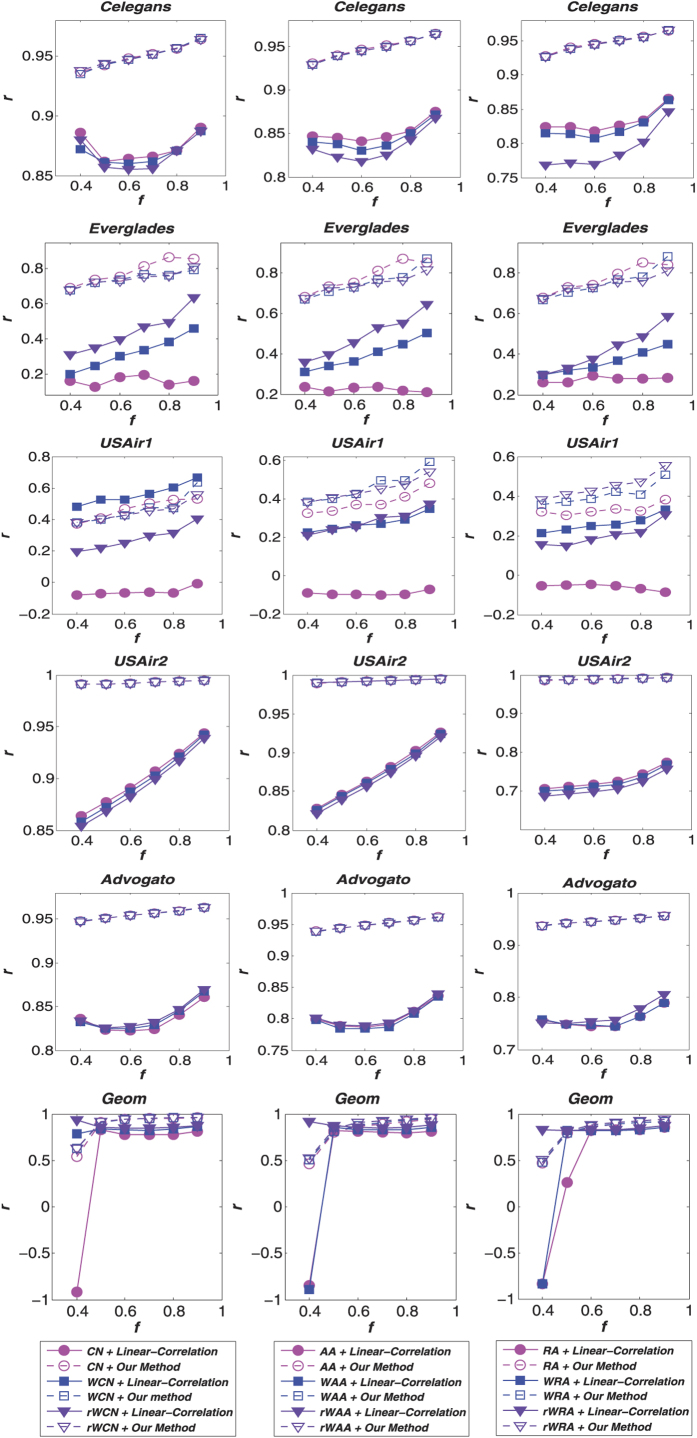
Comparison of the Pearson correlation coefficient (*r*) for weight prediction accuracy under different link prediction algorithms with various training set sizes (*f* denotes the fraction of links from the original network which are used in the training set). The first column of figures shows the results under the *CN*-based methods, the second shows the *AA*-based methods and the third shows the *RA*-based methods.

**Figure 3:**
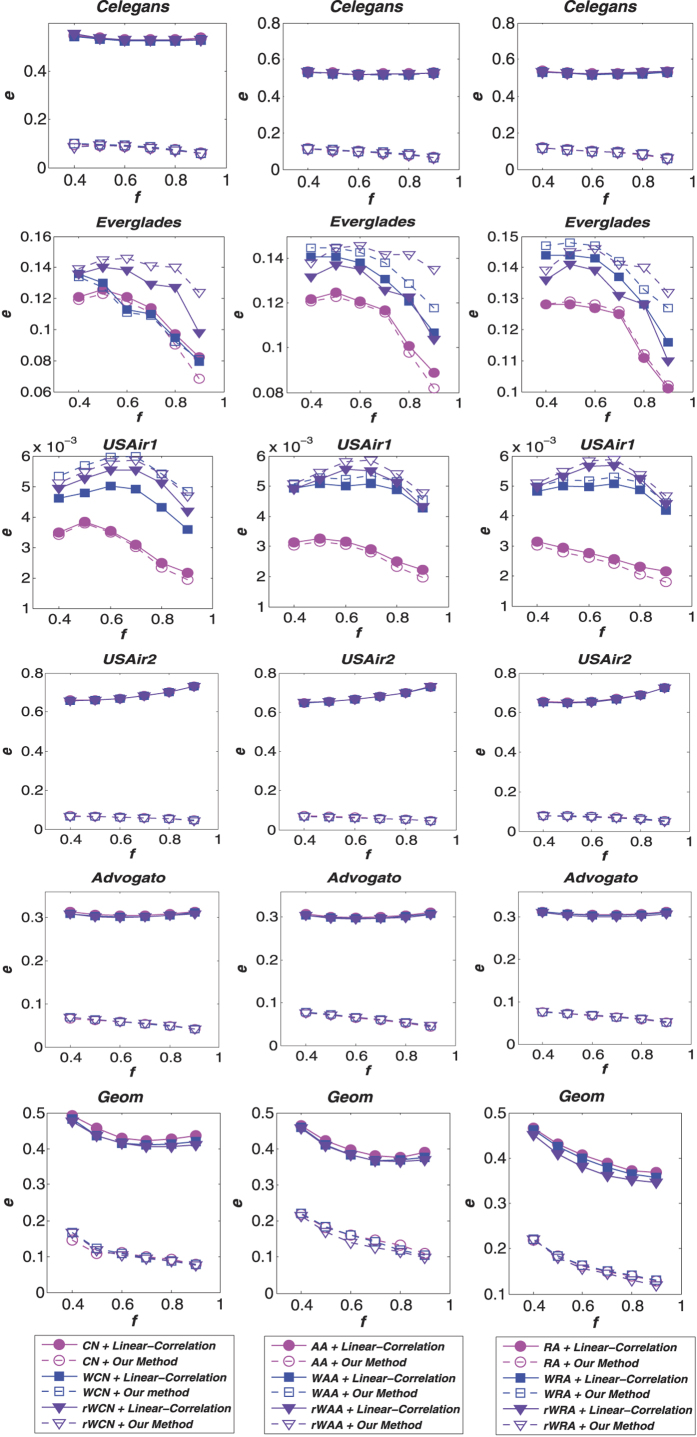
Comparison of the root mean squared error (*e*) for weight prediction accuracy under different link prediction algorithms with various training set sizes (*f* denotes the fraction of links from the original network which are used in the training set). The first column of figures shows the results under the *CN*-based methods, the second shows the *AA*-based methods and the third shows the *RA*-based methods.

Furthermore, to assess the robustness of our method, we also present the accuracy results of weight predictions on different sizes of training set (ranging from 40% to 90%) in Figs 2 and 3. The results demonstrate that the advantages of our method is not sensitive to the density of the network. Because the *CN*-based (*CN*, *WCN* and *rWCN*) indices have similar precisions in link prediction, our weight prediction method yields roughly the same results using these indices, as observed from the nearly identical points in Fig. 2. The same phenomenon also occurs by employing *AA*-based (*AA*, *WAA* and *rWAA*) and *RA*-based (*RA*, *WRA* and *rWRA*) indices. These figures also show that our method outperforms the linear-correlation method in most cases.

## Discussion

In this paper, we explore the problem of weight prediction in weighted networks. A novel weight prediction algorithm which examines the local structure of neighbor set is proposed. To assess the prediction accuracy of our method, empirical experiments are conducted on six real-world networks. The simulation results demonstrate that our method can predict weights much more accurately than the linear-correlation method as measured by the Pearson correlation coefficient and root mean squared error. Furthermore, our method can be used no matter whether the existence of links is missing or not.

## Methods

### Link prediction algorithms

As described above, if the existence of some links is unknown, we need to determine which candidate links are most likely to exist before inferring their weights. Plenty of methods have been proposed to address this link prediction problem. Among them, *Common Neighbors* (CN) is the simplest framework to determine which non-connected node pair is more likely to become connected. Its basic assumption is that two nodes are more likely to form a link if they have more common neighbors. However, CN is limited in that it assumes all common neighbors contribute equally to the connection likelihood. Therefore, several variants of CN have been proposed to remedy this oversight, such as *Adamic*-*Adar* (AA)^36^ and *Resource Allocation* (RA)^15^, which amplify low-degree common neighbors by assigning more weight on them. The precise scores assigned by the different methods are


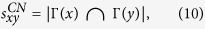



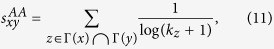



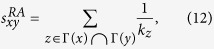


where *k*_*z*_ is defined as the degree of node *z*.

In some real-world networks, links are naturally weighted. Murata and Moriyasu studied the way to extend similarity indices from unweighted networks to weighted networks^27^. Based on this method, the weighted versions of CN, AA and RA (denoted by WCN, WAA and WRA, respectively) are


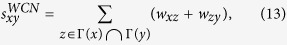



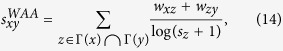



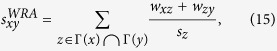


where *w*_*xz*_ is the weight of link (*x*, *z*).

Zhao *et al*. proposed another strategy to generalize similarity indices from unweighted networks to weighted ones based on reliable routes^30^. The weighted versions of CN, AA and RA (denoted as rWCN, rWAA, rWRA, respectively) based on this method are


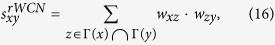



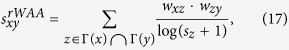



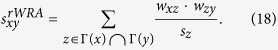


### Weight prediction algorithm for comparison

Now, with the aid of link prediction algorithms, we obtain a set of the candidate links most likely to exist. Because local information is applied for both our method and ref. 30, we compare our performance only with that method. In ref. 30, the authors assumed that the similarity index for link prediction between two unconnected nodes also reflects their interaction strength. Then, inspired by a linear correlation between similarity scores and link weights in many empirical networks, they set the weights of missing links proportional to their similarity scores. Formally, let the weighted adjacency matrix corresponding to the training set *E*^*T*^ and validation set *E*^*V*^ be denoted by *W*^*T*^ and *W*^*V*^, respectively and let *S*^*V*^ be the vector of similarity scores for links in *E*^*V*^. Given the linear correlation mentioned above, we want to find the prediction function *F*(*W*^*T*^) = *λ* · *S*^*V*^, which minimizes the difference between *λ* · *S*^*V*^ and *W*^*V*^, where *λ* is a free parameter. This can be estimated by solving the following optimization problem:


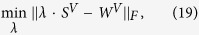


where ||·||_*F*_ is the Frobenius norm, defined as the square root of the sum of the squares of the matrix’s elements. For the sake of brevity, we will call this weight prediction method *linear*-*correlation* in this paper.

### Data description

In this work, we consider six networks to evaluate our new weight prediction method. 1) Celegans^1^: a neural network of the nematode worm *C*. *elegans*, where nodes represent neurons, links join neurons if they have synaptic contacts, and the weight stands for the number of synapses between two neurons. 2) Everglades^37^: a food web network describing carbon exchanges in the Everglades during the wet season, where each node represents a taxon, and an edge denotes that one taxon uses another as food, with link weights representing trophic factors (feeding levels). 3) USAir1^37^: a network of US air transportation, where the weights of links are the frequency of flights between airports. 4) USAir2^38^: a network of flights between US airports in 2010. The weight of a link shows the number of flights between two airports. 5) Advogato^38^: a trust network of the Advogato online community, where nodes represent users of Advogato, links represent trust relationships and weights indicate the trust levels between users. 6) Geom^37^: a collaboration network of researchers in the area of *computational geometry*, where nodes represent authors, links join authors if they have coauthored a paper and weights are the numbers of joint works. To compare the results across different data sets, all link weights are normalized to fall with in the interval [0, 1] as in ref. 30.

## Additional Information

**How to cite this article**: Zhu, B. *et al*. Weight prediction in complex networks based on neighbor set. *Sci. Rep.* **6**, 38080; doi: 10.1038/srep38080 (2016).

**Publisher's note:** Springer Nature remains neutral with regard to jurisdictional claims in published maps and institutional affiliations.

## Figures and Tables

**Table 1:** Comparison of prediction accuracy under the metric of Pearson correlation coefficient for the top-*L* ranked links.

Network\Index	CN	WCN	rWCN	AA	WAA	rWAA	RA	WRA	rWRA
Celegans	0.89	0.887	0.887	0.875	0.872	0.868	0.866	0.863	0.847
	0.964	0.965	0.964	0.965	0.965	0.964	0.965	0.966	0.966
Everglades	0.161	0.459	0.637	0.208	0.501	0.645	0.284	0.449	0.585
	0.858	0.793	0.81	0.85	0.87	0.815	0.84	0.882	0.812
USAir1	−0.0059	0.668	0.403	−0.0711	0.346	0.375	−0.0868	0.332	0.308
	0.531	0.636	0.557	0.481	0.592	0.54	0.382	0.507	0.556
USAir2	0.944	0.942	0.939	0.926	0.924	0.92	0.774	0.768	0.756
	0.995	0.995	0.995	0.995	0.995	0.995	0.994	0.994	0.994
Advogato	0.861	0.867	0.87	0.837	0.835	0.84	0.789	0.79	0.806
	0.963	0.963	0.963	0.962	0.961	0.962	0.956	0.956	0.957
Geom	0.808	0.863	0.876	0.816	0.853	0.878	0.855	0.853	0.871
	0.958	0.961	0.964	0.942	0.948	0.957	0.919	0.919	0.941

In each network, the first row is the results achieved by the linear-correlation method, while the second row shows the accuracy of our method. Each accuracy value is an average over 100 independent random divisions of the links into a training set and a validation set.

**Table 2:** Comparison of prediction accuracy under the metric of root mean squared error for the top-*L* ranked links.

Network\Index	CN	WCN	rWCN	AA	WAA	rWAA	RA	WRA	rWRA
Celegans	0.535	0.529	0.531	0.528	0.524	0.527	0.531	0.53	0.536
	0.0587	0.0595	0.0583	0.0623	0.0629	0.0627	0.0616	0.0616	0.0607
Everglades	0.0821	0.0795	0.0978	0.089	0.107	0.104	0.101	0.116	0.11
	0.0686	0.0806	0.124	0.082	0.118	0.135	0.102	0.127	0.132
USAir1	0.00217	0.00359	0.00419	0.00223	0.00426	0.00433	0.00216	0.00419	0.00441
	0.00194	0.00485	0.00468	0.00197	0.00455	0.00477	0.0018	0.00445	0.00466
USAir2	0.73	0.73	0.731	0.729	0.729	0.73	0.726	0.725	0.724
	0.045	0.0446	0.0442	0.0455	0.045	0.0447	0.053	0.0529	0.0499
Advogato	0.313	0.311	0.309	0.311	0.308	0.306	0.313	0.311	0.307
	0.0408	0.0416	0.0424	0.0439	0.0457	0.0466	0.0506	0.0518	0.0528
Geom	0.435	0.419	0.41	0.39	0.377	0.37	0.369	0.358	0.347
	0.0786	0.077	0.0767	0.11	0.105	0.0978	0.129	0.131	0.12

In each network, the first row is the results achieved by the linear-correlation method, while the second row shows the accuracy of our method. Each accuracy value is an average over 100 independent random divisions of the links into a training set and a validation set.

**Table 3:** Comparison of prediction accuracy under the metric of Pearson correlation coefficient when only the weight information is missing for some links.

Network\Index	Linear-correlation									Ours
	CN	WCN	rWCN	AA	WAA	rWAA	RA	WRA	rWRA	
Celegans	0.203	0.23	0.242	0.238	0.264	0.273	0.271	0.289	0.291	**0**.**379**
Everglades	0.137	0.177	0.415	0.141	0.291	0.455	0.157	0.271	0.406	**0**.**799**
USAir1	0.0403	**0**.**554**	0.305	0.0421	0.24	0.292	0.0712	0.224	0.218	0.513
USAir2	0.259	0.262	0.265	0.255	0.259	0.262	0.2	0.202	0.21	**0**.**378**
Advogato	0.234	0.262	0.289	0.247	0.275	0.303	0.255	0.274	0.314	**0**.**4**
Geom	0.181	0.332	0.45	0.207	0.34	0.496	0.19	0.246	0.517	**0**.**545**

The validation set always contains 10% of the links from the example network. Each accuracy value is an average over 100 independent random divisions of links into a training set and a validation set. In each network, the best performance is emphasized in bold.

**Table 4:** Comparison of prediction accuracy under the metric of root mean squared error when only the weight information is missing for some links.

Network\Index	Linear-correlation									Ours
	CN	WCN	rWCN	AA	WAA	rWAA	RA	WRA	rWRA	
Celegans	0.207	0.206	0.206	0.206	0.204	0.204	0.204	**0.203**	**0.203**	**0.203**
Everglades	0.172	0.171	0.154	0.171	0.164	**0.15**	0.171	0.164	0.156	0.162
USAir1	0.00587	**0.00482**	0.00531	0.00587	0.00571	0.00536	0.00586	0.00573	0.00555	0.00593
USAir2	0.136	0.136	0.136	0.136	0.136	0.136	0.138	0.138	0.137	**0.134**
Advogato	0.107	0.106	0.105	0.106	0.105	0.105	0.106	0.105	**0.104**	0.108
Geom	0.173	0.166	0.158	0.173	0.166	0.153	0.173	0.171	**0.151**	0.185

The validation set always contains 10% of the links from the example network. Each accuracy value is an average over 100 independent random divisions of the links into a training set and a validation set. In each network, the best performance is emphasized in bold.
